# Clinical Prognostic Factors Associated With Relapsing Myelin Oligodendrocyte Glycoprotein Antibody–Associated Disease: A Multicenter Cohort Study

**DOI:** 10.1212/NXI.0000000000200651

**Published:** 2026-08-31

**Authors:** Matthew Human, Christopher G.S. Gilmartin, Athanasios Papathanasiou, Christopher R. Tench, Pakeeran Siriratnam, Chiara Rocchi, Bruno Gran, Milan Hargovan, Ei Zune The, James A. Varley, Saif Huda, Radu Tanasescu

**Affiliations:** 1 Mental Health and Clinical Neuroscience Academic Unit, School of Medicine, NG7 2UH, University of Nottingham, United Kingdom;; 2 Department of Neurology, Nottingham University Hospitals NHS Trust, Queen's Medical Centre, NG7 2UH, United Kingdom;; 3 Department of Neuroscience, Central Clinical School, Monash University, Melbourne, VIC, Australia;; 4 Department of Neurology, Alfred Health, Melbourne, VIC, Australia;; 5 Department of Neurology, Walton Centre NHS Foundation Trust, Liverpool, United Kingdom;; 6 Department of Neurology, Charing Cross Hospital, Imperial College Healthcare NHS Trust, W6 8RF, London, United Kingdom;; 7 Department of Neurology, Leicester Royal Infirmary, Leicester University Hospitals NHS Trust, LE1 5WW, United Kingdom;; 8 Department of Pharmacology and Therapeutics, Institute of Systems, Molecular and Integrative Biology, University of Liverpool, United Kingdom; and; 9 NIHR Nottingham BRC, University of Nottingham, NG7 2UH, United Kingdom.

## Abstract

**Background and Objectives:**

It is currently difficult to accurately predict who, after the index event of myelin oligodendrocyte glycoprotein antibody-associated disease (MOGAD), will develop relapsing disease (R-MOGAD). Several clinical features have been reported as possibly predictive, but none have been validated in clinical practice. We used a prospectively designed analysis to assess a combined model of reported clinical prognosticators for developing R-MOGAD in 101 patients with MOGAD (86% with onset in adulthood) from 3 UK specialist centers.

**Methods:**

A multivariable binary logistic regression model using variables identified from a scoping literature review was fitted in a retrospective clinical data set of patients with MOGAD (Nottingham MS & Neuroinflammation Centre [NUH]), with validation analysis in 2 independent data sets (Walton NMOSD Specialist Centre [WSC]; Imperial College London [ICL]). Secondary analysis investigated time to first relapse using Cox proportional hazards on the combined cohort. Results from the significant variables from the initial analysis were further examined in a meta-analysis of relevant literature studies.

**Results:**

In the Neuroinflammation - Nottingham University Hospitals NHS Trust data set (n = 33), treatment with steroids ≥10 mg for ≥ 3 months after the index event was significantly associated with a lower likelihood of developing R-MOGAD (*p* = 0.006) with sensitivity 83% (95% CI 59–96) and specificity 73% (95% CI 45–92). A persistently positive MOG-IgG status, age at onset, optic neuritis at onset, and sex were not significant predictors of developing R-MOGAD. To assess generalizability, this predictor was tested in 2 independent data sets. In Walton Centre Liverpool (n = 39), not receiving prednisolone ≥10 mg ≥ 3 months was associated with R-MOGAD (OR = 9.7; 95% CI 2.1–45.4; sensitivity 63%; 95% CI 38–84; specificity 85%; 95% CI 62–97). In ICL (n = 29), the association was directionally consistent but inconclusive, with wide confidence intervals crossing unity (OR = 2.7; 95% CI 0.5–13.4; sensitivity 73%; 95% CI 39–94; specificity 50%; 95% CI 26–74). For the combined cohort (n = 101), not receiving prednisolone ≥ 10 mg for ≥3 months was associated with increased odds of developing R-MOGAD (OR = 6.2; 95% CI 2.6–14.8; *p* < 0.0001). Conversely, prednisolone ≥10 mg ≥ 3 months was associated with a lower hazard of relapse (HR = 0.47; 95% CI 0.24–0.91; *p* = 0.024). Median follow-up for monophasic patients was 42 months (IQR: 17.5–64.5).

**Discussion:**

Prednisolone ≥10 mg for ≥ 3 months after the index event was associated with a lower likelihood of relapsing MOGAD. This association was supported in first external cohort and directionally consistent but inconclusive in a second, smaller cohort. Further prospective multicenter studies are required to assess the reproducibility and clinical utility of this association.

## Introduction

Myelin oligodendrocyte glycoprotein antibody-associated disease (MOGAD) is an inflammatory demyelinating disorder of the CNS. MOGAD has either a monophasic or relapsing course^1^ and typically presents with optic neuritis (ON), encephalitis, or myelitis, although unusual cases are reported.^2^ Despite the progress made in diagnosing MOGAD, it is difficult to predict whether a newly diagnosed patient will have a further relapse. The proportion reported in studies of people with MOGAD (pwMOGAD) who develop relapsing MOGAD (R-MOGAD) varies between approximatively 50%^3^ and 80%^4^ and is influenced by the follow-up duration after the index event.^4^ Prognosticating the risk of R-MOGAD is of clinical importance because pwMOGAD with monophasic disease do not require long-term immunosuppression. Neurologic deterioration only occurs during clinical attacks, so preventing relapses is important for reducing disability.^1^

Studies have reported several features, such as clinical phenotype, demographic factors, persistent positive MOG-IgG serostatus, exposure to steroids after the index event correlating with risk of developing early or long-term R-MOGAD^3,5-9^; however, there is a lack of uniform agreement across these studies. To inform generalizability, it is important to use independent patient populations for validation of clinical predictors.

We sought to investigate, using a prospectively planned analysis, the association between previously reported clinical prognostic factors and R-MOGAD. Cross-validation was used to assess the robustness of the derivation analysis. Variables identified as significant in the derivation analysis were subsequently evaluated individually and in 2 independent patient cohorts from 2 different specialist MOGAD centers in the United Kingdom. Further analyses investigated the effect of significant factors on time to first relapse.

## Methods

### Study Design, Setting, and Patients

This study followed a prospectively planned analysis protocol^10^ shared to an online repository in advance of data analysis.

A scoping literature review was undertaken to identify the most suitable factors to include in the multivariable prognostic model for R-MOGAD.^11^ This involved critical appraisal of the number of studies investigating each potential predictor of R-MOGAD and considered the number of studies that found the predictor to have significant value and the number of studies that went against the general consensus of the other studies.^11^ Potential predictors reported by more than 5 studies were included as primary variables. Other variables, where 5 or fewer studies reported a predictive value for R-MOGAD,^11^ were included in a sensitivity analysis.

We collected retrospective clinical data of pwMOGAD 18 years or older of age as of December 2023 at the Nottingham Centre for multiple sclerosis (MS) and Neuroinflammation - Nottingham University Hospitals NHS Trust (NUH). Diagnosis with MOGAD and definition of relapse followed the proposed diagnostic criteria outlined by the International MOGAD panel.^1^ The onset attack of MOGAD as per the proposed diagnostic criteria^1^ was defined as the “index event”. This included pwMOGAD with pediatric-onset disease under follow-up. All pwMOGAD included were seropositive for MOG-IgG by a live cell-based assay^12^ and did not receive maintenance immunotherapy with a steroid-sparing agent. A minimum interval of 30 days was required until first relapse.^1^ Patients without a clear MOGAD diagnosis and those with a confirmed MS diagnosis according to the McDonald Criteria^13^ were excluded. Only pwMOGAD with a minimum follow-up of 12 months were included. All patients from the 3 centers fulfilled MOGAD 2023 diagnostic criteria.^1^

### Statistical Analysis

Statistical analysis methodology was preplanned according to a prospectively defined protocol and endorsed by a senior statistician. Binary logistic regression (BLR) analysis with cross validation was performed using the derivation data set to estimate the ability of the potential predictors to correctly identify relapsing or monophasic disease. Three participants, approximately 10% of the sample, were drawn at random and held out, and the analysis was repeated with 1,000 iterations to estimate prediction accuracy. The R code for the BLR and cross validation is included as eAppendix 1. Sensitivity analysis was performed by stepwise addition of variables into the BLR model and testing predictive accuracy of the model. Individual predictors identified as significant were assessed using the Fisher exact test. Secondary analysis investigated the effect of significant predictors on time to first relapse using Cox proportional hazards on the combined cohort. Additional post hoc sensitivity analyses were performed to explore potential immortal time bias and confounding by indication.

For the identified predictors, we further performed a meta-analysis to contextualize our findings with the current literature. A literature search was completed using the same search string as that of the scoping review to identify eligible studies published up until March 31, 2025, with 2 independent reviewers. Primary studies investigating the identified predictors were identified using appropriate PICO criteria. To ensure homogeneity, the eligible studies were grouped based on statistical analysis and divided into forest plots of those reporting HRs and ORs, respectively, with heterogeneity reported using *I*^2^ statistics. Plots were generated using a peer-reviewed online statistical tool.^14^

### Standard Protocol Approvals, Registrations, and Patient Consents

The collection and analysis of clinical data was approved by the Quality and Safety Committee of NUH NHS Trust (ref.20-241C) and subject to capture in the NUH NMOSD Database (IRAS278736—project approved by West of Scotland Research Ethics Service REC 21/WS/0049). The independent data sets were provided by the NMOSD Specialist Service at the Walton Centre Liverpool (WSC) (study approved by London—Hampstead REC 15/LO/1433, IRAS 180720) and the MOGAD clinic at the Imperial College London (ICL) (Imperial College Healthcare NHS Trust approval: STN_082). The WSC data set included available patient data until March 2024, and the ICL data set available patient data until November 2024. These data sets had not been included in prior publications by the respective groups.^7,15-18^

### Data Availability

Data are available to researchers on request; contact the corresponding author.

## Results

Variables identified from the scoping review^11^ to be included in the combined multivariable prognostic model were prednisolone (corticosteroid) ≥10 mg ≥ 3 months from disease onset, 2 MOG-IgG positive tests ≥ 1 year apart, age at onset, and ON at onset. We excluded ADEM which is a phenotype relevant mainly to childhood onset MOGAD.^1^ Sensitivity analysis included sex, most recent positive MOG-IgG titer from onset (months), prednisolone ≥10 mg duration ≥ 6 months at onset, childhood onset (≤16 years), ethnicity, CSF leukocytes/protein, meningoencephalitis at onset, and viral infection/vaccine preonset; this analysis explored how robust the multivariable prognostic model was to additional variables that might also be predictive.

A total of 33 pwMOGAD from NUH were included in analysis of the predictors (derivation cohort). Subsequently, 2 validation sets of 39 pwMOGAD from WSC and 29 pwMOGAD from ICL were made available. Demographic and clinical characteristics are reported in Tables 1-3 and eTables 1A-2D. The proportion of patients with R-MOGAD was 54.5% in NUH, 48.7% in WSC, and 37.9% in ICL. In the combined cohort (n = 101), median follow-up was 42 months (interquartile range [IQR] 17.5–64.5) for the monophasic group and 77 months (IQR 47–159) for the relapsing cohort.

**Table 1:** Demographic and Clinical Features of Patients From the Nottingham Cohort (NUH)

	Nottingham (NUH) (n = 33)		
	Monophasic	Relapsing	Total
Number of patients (% [n])	45.5 (15)	54.5 (18)	33
Sex (% [n])			
Male	66.7 (10)	44.4 (8)	54.5 (18)
Female	33.3 (5)	55.6 (10)	45.5 (15)
Age at onset (y)			
Mean	34	27	30
Median (IQR)	33 (25–39)	28 (12–36)	29 (22–37)
Childhood onset (<16 y) (% [n])	6.7 (1)	33.3 (6)	21.2 (7)
Non-White ethnicity (% [n])	6.7 (1)	16.7 (3)	12.1 (4)
Follow-up (mo)			
Mean	62	164	117
Median (IQR)	66 (32–83)	138.5 (80–220)	83 (57–163)
Viral infection/vaccine preonset (% [n])	20.0 (3)	22.2 (4)	21.2 (7)
Optic neuritis (ON) at onset (% [n])	53.3 (8)	50.0 (9)	51.5 (17)
Meningoencephalitis at onset (% [n])	0	11.1 (2)	6.1 (2)
First MOG-IgG titer at disease onset (% [n])	86.7 (13)	11.1 (2)	45.5 (15)
Number of MOG-IgG tests (serum or CSF)			
Mean	2	2	2
Median (IQR)	2 (2–3)	2 (1–3)	2 (2–3)
Two MOG-Ab positive tests ≥1y apart (% [n])	6.7 (1)	33.3 (6)	21.2 (7)
Most recent positive MOG-IgG from onset (mo)			
Mean	13	118	70
Median (IQR)	1 (1–11)	115 (55–150)	26 (1–121.5)
CSF leukocytes ≥50 mm^3^/protein ≥450 mg/L at onset (% [n])	26.7 (4)	22.2 (4)	24.2 (8)
Steroids ≥10 mg (duration ≥3 m) at onset (% [n])	73.3 (11)	16.7 (3)	42.4 (14)
Steroids ≥10 mg (duration ≥6m) at onset (% [n])	66.6 (10)	11.1 (2)	36.4 (12)
Early relapse (≤3m) (% [n])	0	16.7 (3)	9.1 (3)
Early relapse (≤6m) (% [n])	0	22.2 (4)	12.1 (4)
Time to first relapse (mo)			
Mean		55	
Median (IQR)		28 (6–72)	

Abbreviations: IQR = interquartile range; NUH = Neuroinflammation-Nottingham University Hospitals NHS trust.

**Table 2:** Demographic and Clinical Features of Patients From the Liverpool Cohort (WSC)

	Liverpool (WSC) (n = 39)		
	Monophasic	Relapsing	Total
Number of patients (% [n])	51.3 (20)	48.7 (19)	39
Sex (% [n])			
Male	45.0 (9)	47.4 (9)	46.2 (18)
Female	55.0 (11)	52.6 (10)	53.8 (21)
Age at onset (y)			
Mean	34	29	32
Median (IQR)	29.5 (22.5–45)	28 (19–35)	29 (22–40)
Childhood onset (<16 y) (% [n])	0	21.1 (4)	10.3 (4)
Non-White ethnicity (% [n])	5.0 (1)	15.8 (3)	10.3 (4)
Follow-up (mo)			
Mean	26	101	62
Median (IQR)	21 (14.5–39.5)	62 (33–111)	39 (21–62)
Viral infection/vaccine preonset (% [n])	35.0 (7)	15.8 (3)	25.6 (10)
Optic neuritis (ON) at onset (% [n])	85.0 (17)	57.9 (11)	71.8 (28)
Meningoencephalitis at onset (% [n])	0	5.3 (1)	2.6 (1)
First MOG-IgG titer at disease onset (% [n])	85.0 (17)	26.3 (5)	56.4 (22)
Number of MOG-IgG tests (serum or CSF)			
Mean	3	4	4
Median (IQR)	3 (2.5–3.5)	4 (2–5)	3 (2–5)
Two MOG-Ab positive tests ≥1y apart (% [n])	30.0 (6)	84.2 (16)	56.4 (22)
Mean	13	90	50
Median (IQR)	6.5 (1–14)	54 (24–101)	21 (5–54)
CSF leukocytes ≥50 mm^3^/protein ≥450 mg/L at onset (% [n])	20.0 (4)	15.8 (3)	17.9 (7)
Steroids ≥10 mg (duration ≥3m) at onset (% [n])	85.0 (17)	36.8 (7)	61.5 (24)
Steroids ≥10 mg (duration ≥6m) at onset (% [n])	75.0 (15)	15.8 (3)	46.2 (18)
Early relapse (≤3m) (% [n])	0	10.5 (2)	5.1 (2)
Early relapse (≤6m) (% [n])	0	21.1 (4)	10.3 (4)
Time to first relapse (mo)			
Mean		29	
Median (IQR)		12 (6–42)	

Abbreviations: IQR = interquartile range; WCL = Walton Centre Liverpool.

**Table 3:** Demographic and Clinical Features of Patients From the London Cohort (ICL)

	London (ICL) (n = 29)		
	Monophasic	Relapsing	Total
Number of patients (% [n])	62.1 (18)	37.9 (11)	29
Sex (% [n])			
Male	33.3 (6)	45.5 (5)	37.9 (11)
Female	66.7 (12)	54.5 (6)	62.1 (18)
Age at onset (y)			
Mean	35	38	36
Median (IQR)	35.5 (28–40)	36 (24–59)	36 (25.5–45)
Childhood onset (<16 y) (% [n])	11.1 (2)	9.1 (1)	10.3 (3)
Non-White ethnicity (% [n])	50.0 (9)	45.5 (5)	48.3 (14)
Follow-up (mo)			
Mean	55	73	62
Median (IQR)	66 (53–70)	62 (39–90)	66 (42–76)
Viral infection/vaccine preonset (% [n])	11.1 (2)	9.1 (1)	10.3 (3)
Optic neuritis (ON) at onset (% [n])	55.6 (10)	72.7 (8)	62.1 (18)
Meningoencephalitis at onset (% [n])	16.7 (3)	27.3 (3)	20.7 (6)
First MOG-IgG titer at disease onset (% [n])	83.3 (15)	55.5 (6)	72.4 (21)
Number of MOG-IgG tests (serum or CSF)			
Mean	3	5	3
Median (IQR)	2.5 (2–3)	4 (3–6)	3 (2–4)
Two MOG-Ab positive tests ≥1y apart (% [n])	33.3 (6)	72.7 (8)	48.3 (14)
Mean	35	45	39
Median (IQR)	47.5 (3–56)	44 (6–70)	45 (3.5–60)
CSF leukocytes ≥50 mm^3^/protein ≥450 mg/L at onset (% [n])	22.2 (4)	36.4 (4)	27.6 (8)
Steroids ≥10 mg (duration ≥3m) at onset (% [n])	50.0 (9)	27.3 (3)	41.4 (12)
Steroids ≥10 mg (duration ≥6m) at onset (% [n])	11.1 (2)	0	6.9 (2)
Early relapse (≤3m) (% [n])	0	18.2 (2)	6.9 (2)
Early relapse (≤6m) (% [n])	0	36.4 (4)	13.8 (4)
Time to first relapse (mo)			
Mean		13	
Median (IQR)		9 (4–24)	

Abbreviations: ICL = Imperial College London; IQR = interquartile range.

The duration of follow-up for the 3 data sets is presented in detail in Table 1. NUH had the longest median duration (83 months), followed by ICL (66 months) and WSC (39 months). The median duration of follow-up for monophasic patients was similar in NUH and ICL (66 months) and shorter in WSC (21 months). The median duration of follow-up for relapsing patients was similar in ICL and WSC (62 months) and longer in NUH (138.5 months).

In NUH, 7 of 33 (21.2%) patients; in WSC 4 of 39 (10.3%); and in ICL 3 of 29 (10.3%) had disease onset in childhood (≤16 years).

The proportion of pwMOGAD who received prednisolone ≥10 mg ≥ 3 m from disease onset was 42.4%, 61.5%, and 41.4% in NUH, WSC, and ICL, respectively. No pwMOGAD in the derivation cohort experienced a grade >3 adverse effect as per the Common Terminology Criteria for Adverse Events classification.^19^

The BLR model identified prednisolone ≥10 mg ≥ 3 m from disease onset as the only significant variable, with a negative association with R-MOGAD (logarithmic odds −2.68, 95% CI –4.62 to −0.74). Two MOG-IgG positive tests ≥ 1 year apart (*p* = 0.068), age at onset (*p* = 0.226), and ON at onset (*p* = 0.529) were not significant predictors of R-MOGAD (Table 4).

**Table 4:** Results of Multivariable Binary Logistic Regression Analysis Showing the Estimates and Significance for Each Variable Within the Model to Predict the Outcome of R-MOGAD

	Logarithmic odds for R-MOGAD	Standard error	*p* Value
Prednisolone (corticosteroids) ≥10 mg duration ≥3 m from onset	−2.68	0.97	0.006
Two MOG-IgG positive tests ≥1y apart	2.52	1.38	0.068
Age at onset	−0.04	0.03	0.226
ON at onset	−0.59	0.94	0.529

One thousand iterations of cross-validation showed that disease course (monophasic vs relapsing) could on average be correctly predicted in 85% ± 15% of held-out cases; this was unchanged when the number of held out participants was increased to 5 and 10 (of 33). As only 1 predictor was identified as significant, the multivariable prognostic model was simplified to a 2-by-2 contingency table describing the association between not receiving prednisolone ≥10 mg ≥ 3 m with R-MOGAD. The resultant model found that not receiving prednisolone ≥10 mg ≥ 3 m was associated with R-MOGAD (OR = 13.8, 95% CI 2.5–74.3) with sensitivity 83% (95% CI 59–96) and specificity 73% (95% CI 45–92) in the derivation data set (eTable 3).

Sex, childhood onset (≤16 years), ethnicity, CSF leukocytes/protein, meningoencephalitis at onset, and viral infection/vaccine preonset did not produce additional significant results or remove the significance of prednisolone ≥10 mg ≥ 3 m from onset when inputted individually into the model.

The association between prednisolone ≥10 mg ≥ 3 m and R-MOGAD was evaluated in 2 independent cohorts. In WSC (n = 39), not receiving prednisolone ≥10 mg ≥ 3 m was associated with R-MOGAD (OR = 9.7, 95% CI 2.1–45.4), with sensitivity 63% (95% CI 38–84) and specificity 85% (95% CI 62–97) (eTable 4). In ICL (n = 29), the association was directionally consistent but statistically inconclusive (OR = 2.7, 95% CI 0.5–13.4), with wide confidence intervals crossing unity and lower specificity (sensitivity 73%, 95% CI 39–94; specificity 50%, 95% CI 26–74) (eTable 5). For the combined cohort (n = 101), not receiving prednisolone ≥10 mg ≥ 3 m had an OR = 6.2 (95% CI 2.6–14.8; *p* < 0.0001), sensitivity 73% (95% CI 58–85), and specificity 70% (95% CI 56–82) for R-MOGAD (eTable 6). Given the inclusion of patients with childhood-onset disease, an adult-onset–only sensitivity analysis was performed. In patients with adulthood onset (≥17 years; n = 87), not receiving prednisolone ≥10 mg ≥ 3 m remained associated with increased odds of R-MOGAD (OR = 6.1, 95% CI 2.4–15.5; *p* = 0.0002), with sensitivity 70% (95% CI 53–84) and specificity 72% (95% CI 58–84) (eTable 7).

A secondary analysis was performed to investigate the effect of prednisolone ≥10 mg ≥ 3 m on time to first relapse using the Cox proportional hazards model, using the combined cohort (n = 101). This demonstrated that prednisolone ≥10 mg ≥ 3 m was associated with a lower hazard of first relapse (HR 0.47, 95% CI 0.24–0.91; *p* = 0.024). This provides further confidence, particularly regarding differing follow-up durations between monophasic and relapsing groups.

To further explore the potential effect of immortal time bias, we reviewed the 8 patients who experienced a first relapse within 3 months of disease onset. Three of these patients subsequently fulfilled the definition of prednisolone ≥ 10 mg ≥ 3 m but had not completed this exposure period before their first relapse. It is important that all 8 patients continued to experience relapses beyond 3 months from onset and therefore remained classified as having R-MOGAD after application of the 3-month landmark.

A post hoc 3-month landmark analysis was subsequently performed. Prednisolone exposure remained associated with a lower hazard of first relapse, with a similar direction and magnitude of association with the primary analyses, although statistical significance was not retained (HR 0.55, 95% CI 0.28–1.06; *p* = 0.074).

To explore potential confounding by indication, additional analyses were performed in the WSC cohort, where EDSS at the index event was available. The median EDSS score was 4 (IQR 3–7) for pwMOGAD who subsequently received prednisolone ≥10 mg ≥ 3 m vs 3 (IQR 3–4) for pwMOGAD who did not receive prolonged corticosteroid treatment. However, the correlation between EDSS at the index event and prednisolone ≥10 mg ≥ 3 m was weak (correlation coefficient 0.16). An EDSS-adjusted Cox proportional hazards model examining the association between prednisolone ≥ 10 mg ≥ 3 m and R-MOGAD in the WSC cohort yielded a hazard ratio (HR) of 0.58 (95% CI 0.21–1.64; *p* = 0.305).

To further contextualize the observed association between corticosteroid exposure and relapse risk, we conducted a meta-analysis assessing the relationship between steroid duration at disease onset, and the risk of developing R-MOGAD. An additional literature search was performed using the same search string as that of the scoping review to identify further eligible studies published up until March 31, 2025 (eFigure 1), with 2 reviewers assessing results. Criteria for both searches are described in eTable 8. Primary studies investigating steroids as a predictor of R-MOGAD were identified using predefined PICO criteria (eTable 9).

Across both searches, 7 eligible studies were identified all of which were retrospective cohort studies. A summary of the included and excluded literature is provided in eTables 10 and 11, respectively. The 2 eligible studies included in the initial scoping review were Huda et al.^7^ and Nosadini et al.,^20^ which both found pwMOGAD receiving steroid treatment ≥ 1 month to have significantly lower odds of relapse. Five articles published after the scoping review that met the PICO criteria were identified.^5,6,21-23^ Four of the studies identified presented results as HRs,^5,6,21,22^ and these were incorporated with the results of our Cox proportional hazards model on the combined data set. Three of these studies investigated a minimum treatment duration of 3 months^5,6,22^ and the remaining study 1 month,^21^ so 2 forest plots were generated with a minimum exposure of 3 months (eFigure 2) and 1 month (eFigure 3), respectively. The remaining 3 studies identified from the literature searches all reported the association between receiving steroids and developing R-MOGAD as ORs.^7,20,23^ Two of these studies analyzed a minimum duration of 1 month^7,20^ and the other 7 months,^23^ so a third forest plot was generated for the pooled OR of receiving steroid treatment ≥ 1 month (eFigure 4).

To pool these with our results, a 2-by-2 contingency table was generated testing the association between R-MOGAD and receiving steroids ≥ 10 mg ≥ 3 months instead of not receiving steroids in our combined cohort. This again found a significant negative association between receiving steroids ≥10 mg ≥ 3 months and R-MOGAD (OR 0.16, 95% CI 0.07–0.38; *p* < 0.0001). For the studies reporting ORs,^7,20,23^ steroid treatment ≥ 1 month was associated with lower odds of R-MOGAD (pooled OR 0.16, 95% CI 0.09–0.29, *I*^2^ 0.0%) (eFigure 4).

The analysis of the pooled data of the studies investigating steroid treatment ≥ 3 months using Cox proportional hazards^5,6,22^ also suggests that this is associated with a lower hazard of relapse, but this was not significant (pooled HR 0.57, 95% CI 0.24–1.35, *I*^2^ 80.8%) (eFigure 2). Similarly, a minimum duration of 1 month of steroid treatment associated with a lower hazard of relapse but was not significant (pooled HR 0.64, 95% CI 0.36–1.14, *I*^2^ 74.6%) (eFigure 3).

## Discussion

This retrospective cohort study with a preplanned analysis found that prednisolone ≥10 mg treatment for ≥3 months after the index event was associated with a lower likelihood of R-MOGAD. This association was supported in WSC, directionally consistent but inconclusive in ICL, and associated with a lower hazard of first relapse in the combined cohort (n = 101). This is in keeping with previous evidence reporting an association between corticosteroid use and lower MOGAD relapse risk^7,8,20,23,24^ and recent data supporting the use of corticosteroids ≥12.5 mg ≥ 3 months to delay time to first relapse in adults with MOGAD.^5^ This is in contrast to recent data in pediatric pwMOGAD, where maintenance steroids were associated with a higher estimated relapse risk.^6^

To contextualize our findings, we performed a meta-analysis of 8 studies commenting on corticosteroid treatment and relapse risk. Steroid treatment for ≥ 1 month was associated with lower odds of R-MOGAD (pooled OR 0.16, 95% CI 0.09–0.29; *p* < 0.01), whereas pooled hazard ratio analyses suggested a similar direction of association that did not reach statistical significance.

The only study investigating corticosteroid treatment ≥ 3 months and time to first relapse using Cox proportional hazards, alongside our combined cohort, was Trewin et al.,^5^ who reported a significant association between maintenance corticosteroids and delayed first relapse (HR 0.12, 95% CI 0.03–0.44). Their cohort had a median follow-up of 74.4 months and identified a minimum effective dose of 12.5 mg/day using Simon-Makuch plots, which may partly explain the stronger effect size observed.

By contrast, Virupakshaiah et al.^6^ reported a nonsignificant trend toward increased relapse risk despite corticosteroid treatment (HR 1.76). This was a pediatric-only cohort and may not be directly comparable.

Studies reporting odds ratios^7,20,23^ consistently found prolonged corticosteroid exposure to be associated with monophasic disease, in keeping with the results of our contingency-table analyses. However, these cohorts differed substantially in age composition and corticosteroid exposure definitions, which may affect generalizability.

This post hoc meta-analysis should be interpreted cautiously. Although studies were grouped according to statistical methodology and treatment duration where possible, the included cohorts differed in age composition, corticosteroid exposure thresholds, outcome definitions, and clinical practice. This was reflected by substantial heterogeneity in the pooled hazard ratio analyses (*I*^2^ 74.6%–80.8%), whereas heterogeneity was absent in the pooled odds ratio analysis (*I*^2^ 0%). Given the limited number of available studies addressing corticosteroid exposure in MOGAD, further stratification was not feasible. Consequently, the meta-analysis was intended primarily to provide context for the present findings rather than a definitive estimate of treatment association.

A strength of this study is the pragmatic, prospective analysis approach taken, with its protocol published before data analysis. We present a long mean follow-up (58 m for the combined cohort, 117 m for the derivation data set), compared with other studies.^7,20-22,25^ It is important that we report a longer median follow-up duration for monophasic patients than those in the meta-analysis (eTable 9) and other recent studies.^5,7,20,21,26^ A further strength is the rigorous approach taken to appraise the literature for identifying the most suitable predictors to be included in the model while limiting partiality.

The derivation cohort was modest in size relative to the number of candidate predictors evaluated and therefore may be susceptible to overfitting and instability of coefficient estimates. Although candidate predictors were selected a priori through a literature-informed protocol developed before analysis, the protocol did not explicitly prespecify reduction of the multivariable model to a single predictor for subsequent evaluation. Consequently, the possibility of optimism arising from model reduction cannot be excluded. External validation in 2 independent specialist-center cohorts mitigates, but does not eliminate, this risk.

The ICL cohort provided an inconclusive external evaluation of the steroid association. Although the direction of effect was consistent with NUH and WSC, the CI was wide and crossed unity, and specificity was lower than in the other cohorts. This likely reflects, at least in part, the small sample size, fewer relapsing patients, and potential population-specific differences in referral patterns, follow-up, and corticosteroid prescribing practices. Therefore, only 1 of the 2 external cohorts provided clear statistical support for the association, and further larger prospective cohorts are required to determine the reproducibility and generalizability of this finding.

In the derivation cohort, the mode number of MOG-IgG titers was only 2 (in 45%), and 7 pwMOGAD (21%) had only 1 titer result available. Furthermore, 18 of 33 (55%) pwMOGAD did not receive MOG-IgG testing at disease onset. Persistently positive MOG-IgG titers have been shown to be predictive of relapse compared with seronegative conversion,^21,27-30^ but our study and others^31-35^ did not find a significant association. Our results show a trend to significance and are likely explained by sample size and type II error.

Age and ON were not significant predictors either, which corresponds to the heterogeneity in the literature regarding these predictors.^5-7,9,20-22,32,35-37^ In our sensitivity analysis, sex did not influence the robustness of the BLR model. Female sex has been associated with relapse in some studies^6,7,23,35,38^ but not others,^5,20-22,32,39^ and evidence remains heterogeneous. Similarly, non-White ethnicity has been reported to be associated with a higher risk of relapse by some studies^6,36,40^ but not others.^20,22,39^

The duration of follow-up of monophasic patients affects the likelihood of developing R-MOGAD. Although the cumulative probability of relapse increases with longer follow-up, the underlying hazard is not linear but front-loaded. Most first relapses occur early, with continued accrual over time, as demonstrated in large cohorts. In a UK multicenter study, relapse risk increased from 31.7% at 4 years to 36.3% at 8 years,^39^ whereas in another cohort, the median time to first relapse was 11.5 months, with the majority occurring within the first year.^7^ Late first relapses remain recognized.^4^

In this context, the duration of follow-up in our monophasic cohort (median 42 months, IQR 17.5–64.5), with particularly long follow-up in the derivation NUH cohort (median 66 months), extends well into the period during which first relapses are commonly observed and is likely to capture a clinically relevant window of relapse risk. Consistent with this, follow-up in our cohorts is comparable with, or longer than, that reported in recent studies (eTable 9). A recent retrospective multicenter validation study of the MOG-AR score^26^ similarly identified immunosuppressive treatment after the first event as the only factor significantly associated with a lower relapse risk in MOGAD; however, this study reported a shorter median follow-up for monophasic patients (34.4 months, IQR 21.6–57.2) than in our cohort. Furthermore, using study-level follow-up data for monophasic and relapsing MOGAD from 48 studies included in a recent systematic review and meta-analysis of laboratory biomarkers and relapse risk,^27^ we calculated a weighted median follow-up of 29 months for monophasic patients with MOGAD across these 48 studies, which is substantially shorter than in our cohort (29 vs 42 months).

Taken together, these findings indicate that follow-up in our cohort is comparable with, and in many cases exceeds, that reported in contemporary studies. In a 2026 systematic review,^27^ many individual cohorts reporting comparable follow-up data were modest in size, and our combined cohort of 101 patients lies at the upper end of published study sizes.

The current practice in the NUH MOGAD clinic is to follow-up all MOGAD cases even if they remain monophasic; however, this did not apply to historical cases. This led to the limitation of a discrepancy in follow-up duration between monophasic and relapsing forms of MOGAD in this retrospective data set. This difference in follow-up was observed in WSC but not ICL, where monophasic patients were followed up for longer than relapsing patients (median 66 vs 62 months). It is known that some pwMOGAD have late first relapses^4,41^ and within our NUH R-MOGAD cohort 38.9% (7/18) had their first relapse > 5 years after their index event (eFigure 5). Historical cases are also prone to referral bias and difficulties in ascertaining relapse retrospectively. This prompted a Cox regression analysis which produced results commensurate with the planned analysis, avoiding any assumptions regarding long-term outcomes. Most monophasic patients in the derivation cohort had > 5 years of follow-up, which is a strong time frame to be confident in this classification. However, median follow-up was shorter in both WSC (39 months) and ICL (66 months), meaning late first relapses beyond this period in the validation data sets cannot be ruled out. This limitation is not unique to our study; in their recently published meta-analysis, Andersen et al.^27^ found that 25% (12/48) of studies reporting follow-up duration had a significantly longer duration in relapsing compared with monophasic groups.^27^

A further limitation relates to the potential for immortal time bias arising from the definition of prolonged corticosteroid exposure. Patients who relapsed before completing 3 months of corticosteroid treatment could not fully contribute to the exposed group before their first relapse. To explore this, we reviewed all patients who experienced a first relapse within 3 months of disease onset and performed a post hoc 3-month landmark analysis. All patients who relapsed within 3 months remained classified as having R-MOGAD after application of the landmark, indicating that relapse classification was not altered by this approach. Furthermore, the landmark analysis yielded an association of similar direction and magnitude to the primary analyses; however, statistical significance was not retained. These findings should therefore be interpreted cautiously and indicate that although the observed association was not solely dependent on events occurring before the landmark period, the potential contribution of immortal time bias to the observed association cannot be excluded.

To maximize eligibility and follow-up, patients with both childhood-onset and adulthood-onset MOGAD were included. In NUH, WSC, and ICL, 21.2%, 10.3%, and 10.3% of pwMOGAD had disease onset in childhood, respectively, of whom 85.7%, 100.0%, and 33.3% had R-MOGAD. We acknowledge a selection bias in some centers because relapsing patients with childhood onset were more likely to be followed up into adulthood than monophasic patients. In addition, pediatric-onset MOGAD may differ from adult-onset disease in clinical phenotype, relapse patterns, and treatment practices, including corticosteroid prescribing and tapering approaches. The variation in relapse rates among childhood-onset patients across cohorts may therefore have contributed to intercohort heterogeneity independent of corticosteroid exposure. To explore this potential source of bias, we performed a sensitivity analysis restricted to patients with adulthood-onset disease in which not receiving prednisolone ≥ 10 mg ≥ 3 m remained significantly associated with an increased risk of developing R-MOGAD. However, the study was not powered to evaluate treatment associations separately in pediatric-onset and adult-onset MOGAD, and effect modification by age at onset cannot be excluded. Future larger age-stratified cohorts are needed to determine whether the association between corticosteroid exposure and relapse risk differs between pediatric-onset and adult-onset disease.

Confounding by indication remains an important limitation. The severity and extent of recovery from the index event may influence the clinician's decision regarding the duration and dose of corticosteroid treatment. In the WSC cohort, patients who subsequently received prolonged corticosteroid treatment had numerically higher EDSS scores at the index event than those who did not receive prolonged corticosteroids. However, formal assessment demonstrated only a weak correlation between EDSS and prolonged corticosteroid exposure (correlation coefficient 0.16). Furthermore, adjustment for EDSS in the WSC cohort did not provide evidence that the observed association was explained by EDSS alone. These analyses were limited to the WSC cohort because EDSS at the index event was not available for NUH or ICL, making this a primary limitation. Residual confounding by indication therefore cannot be excluded and should be addressed in future prospective studies with systematic collection of attack severity, recovery, treatment indication, corticosteroid dose, and taper duration.

We did not include imaging findings in our model. Although MRI and OCT are valuable for diagnosis and disease differentiation,^42,43^ their predictive role for relapse is not yet established.^44^

Although this study evaluated multiple candidate clinical prognostic factors, prolonged corticosteroid exposure was the only variable that demonstrated an association with relapse risk in the derivation cohort and remained directionally consistent across the validation cohorts. Accordingly, the findings should be interpreted primarily in prognostic association and clinical relevance rather than as a fully developed prediction model. Future prognostic models for relapsing MOGAD will likely require integration of additional clinical, radiologic, immunologic, and treatment-related variables within larger prospective data sets.

Here, we report, using a preplanned analytical approach, that prednisolone ≥10 mg for ≥3 months after the index MOGAD event was associated with a lower likelihood of R-MOGAD. Given the observational nature of the study and the potential for residual confounding, these findings should be interpreted as prognostic associations rather than evidence of treatment effect. Prospective multicenter studies are required to determine whether prolonged corticosteroid treatment influences relapse risk in MOGAD.

## Glossary

BLR: binary logistic regression
ICL: Imperial College London
IQR: interquartile range
MOGAD: myelin oligodendrocyte glycoprotein antibody-associated disease
MS: multiple sclerosis
NUH: Neuroinflammation-Nottingham University Hospitals NHS Trust
ON: optic neuritis
pwMOGAD: people with MOGAD
R-MOGAD: relapsing disease-MOGAD
WSC: Walton Centre Liverpool
